# SELF-CARE AS A FORTITUDE FACTOR AGAINST COMPASSION FATIGUE: LESSONS LEARNED FROM COVID-19

**DOI:** 10.1093/geroni/igae098.3708

**Published:** 2024-12-31

**Authors:** Cathy Scott, Junrong Shi, Linda Samuel

**Affiliations:** University of Tennessee at Chattanooga, Chattanooga, Tennessee, United States; University of Tennessee Chattanooga, Chattanooga, Tennessee, United States; North Carolina A & T State University, North Carolina A & T State University, North Carolina, United States

## Abstract

Gerontology practitioners did not receive the national attention as others, but they were on the frontlines during the COVID-19 pandemic caring for older adults, one of our nation’s most vulnerable populations. They helped older adults manage health complications, isolation and navigate limited services. This mixed-methods study examined self-care among gerontology practitioners. Qualitative data were collected through semi-structured interviews with 13 gerontological practitioners. The thematic content analysis demonstrated the overall well-being of gerontology practitioners during the COVID-19 pandemic including fear, physical exhaustion, and anxiety coupled with an unsupportive workplace are associated with compassion fatigue. Gerontology practitioners described spiritual/faith practices, leisure activities, and the support of family as self-care activities that sustained them and contributed to their constancy while serving older adults. Quantitative data were collected using an online survey. The statistical analysis with a small sample (N= 37) showed that gerontological practitioners engaged in self-care activities (M=31.9, SD=4.83, range=22-41), measured by a 10-item Likert-scale (Cronbach alpha=0.60). They showed a medium level of compassion fatigue (M=29.7, SD=7.86, range:16-45), measured by a 10-item Likert-scale (Cronbach alpha=0.88). The linear regression showed a strong negative correlation between self-care and compassionate fatigue (b=-0.8, p< 0.01) controlling for age, race, professional field, and years of working with older people (gender is not included with only two males). Findings suggest self-care as a protective factor against compassion fatigue and the need for increased administrative support/resources, communication, and recognition of the psychological impact on clients and practitioners, especially for agencies working with chronic and terminally ill clients during crisis situations.

